# Reviewing Interventions against Enterobacteriaceae in Broiler Processing: Using Old Techniques for Meeting the New Challenges of ESBL* E. coli*?

**DOI:** 10.1155/2018/7309346

**Published:** 2018-10-23

**Authors:** Michaela Projahn, Ewa Pacholewicz, Evelyne Becker, Guido Correia-Carreira, Niels Bandick, Annemarie Kaesbohrer

**Affiliations:** German Federal Institute for Risk Assessment, Diedersdorfer Weg 1, 12277 Berlin, Germany

## Abstract

Extended-spectrum beta-lactamase- (ESBL-) producing Enterobacteriaceae are frequently detected in poultry and fresh chicken meat. Due to the high prevalence, an impact on human colonization and the spread of antibiotic resistance into the environment is assumed. ESBL-producing Enterobacteriaceae can be transmitted along the broiler production chain but also their persistence is reported because of insufficient cleaning and disinfection. Processing of broiler chickens leads to a reduction of microbiological counts on the carcasses. However, processing steps like scalding, defeathering, and evisceration are critical concerning fecal contamination and, therefore, cross-contamination with bacterial strains. Respective intervention measures along the slaughter processing line aim at reducing the microbiological load on broiler carcasses as well as preventing cross-contamination. Published data on the impact of possible intervention measures against ESBL-producing Enterobacteriaceae are missing and, therefore, we focused on processing measures concerning Enterobacteriaceae, in particular* E. coli* or coliform counts, during processing of broiler chickens to identify possible hints for effective strategies to reduce these resistant bacteria. In total, 73 publications were analyzed and data on the quantitative reductions were extracted. Most investigations concentrated on scalding, postdefeathering washes, and improvements in the chilling process and were already published in and before 2008 (n=42, 58%). Therefore, certain measures may be already installed in slaughterhouse facilities today. The effect on eliminating ESBL-producing Enterobacteriaceae is questionable as there are still positive chicken meat samples found. A huge number of studies dealt with different applications of chlorine substances which are not approved in the European Union and the reduction level did not exceed 3 log10 values. None of the measures was able to totally eradicate Enterobacteriaceae from the broiler carcasses indicating the need to develop intervention measures to prevent contamination with ESBL-producing Enterobacteriaceae and, therefore, the exposure of humans and the further release of antibiotic resistances into the environment.

## 1. Introduction

Enterobacteriaceae that produce extended-spectrum beta-lactamases (ESBLs) are a challenging problem in human and veterinary medicine due to the limitations of the treatment options against infections caused by these resistant bacteria. In the beginning, ESBL-producing Enterobacteriaceae were only linked to human infections in hospitals. Nowadays, they are found to be widespread as intestinal gut colonizers in healthy humans as well as in animals and were also isolated from environmental samples like wastewater or fresh surface waters [1–3]. As also farm animals, especially broiler chickens, are affected by ESBL-producing* E. coli* (EEC), transmission via the food production chain from animals to humans is speculated [4, 5]. Therefore, many studies were conducted to investigate the dissemination of these resistant bacteria in the broiler production. EEC were found in broiler breeder chickens and also in samples from broiler fattening farms even though there was no antibiotic treatment [6–11]. Moreover, in some studies broiler chickens were found to be already colonized with resistant bacteria in their first days of life [9]. Transmission investigations identified potential (pseudo-)vertical transmission routes from (grand-)parent flocks to their offspring where the hatcheries played an important role in further spreading the resistant* E. coli* strains into the fattening flocks [12–15]. In addition, insufficient cleaning and disinfection procedures support the circulation of EEC on the farms and between subsequently fattened flocks [16, 17].

Further investigations focused on the occurrence of EEC in chicken retail meat and chicken food products. Proportions of up to 90% of EEC-positive samples were reported in some European studies [18–20]. Therefore, chicken meat is considered as a potential source of the transmission to humans. Insufficient kitchen hygiene in private households or hospitals might contribute to cross-contamination or transmission to humans [21, 22] even though the amount of detectable EEC on chicken filet or neck skin samples seemed to be low (range of 1 to 3.18 log10 CFU/g) [23, 24].

Processing of broiler carcasses in slaughterhouses is very likely a critical point in the contamination of raw chicken meat. On one hand, EEC frequently occur as intestinal colonizers in broiler chickens and, therefore, fecal contamination during evisceration of carcasses might be possible [25, 26]. On the other hand, broiler chickens are obviously visibly contaminated on the skin and feathers with a mixture of feces and litter before entering the slaughterhouse [27, 28]. Unfortunately, there are only limited data on the occurrence of EEC during the processing of broiler chickens in the slaughterhouse. In the study of Pacholewicz et al., they found an overall reduction of EEC on broiler carcasses during processing [29]. However, in some of the investigated batches more than 2 log EEC per carcass could be still detected after chilling. Therefore, intervention measures against these resistant bacteria in slaughterhouse facilities are needed to further reduce or even eliminate EEC from broiler processing plants and, therefore, also from chicken retail meat.

A lot of research was conducted to investigate intervention measures against* Salmonella* sp. or* Campylobacter* sp. in slaughterhouses [30] but to the best of our knowledge none of the studies examined the respective interventions concerning the usefulness to eradicate EEC. However, some studies concerning* Salmonella* sp. or* Campylobacter* sp. also determined the counts of* E. coli*, coliforms, or total Enterobacteriaceae in their samples. These data might give an indication of the effectiveness of a respective intervention against EEC in the processing plants as Enterobacteriaceae, in particular* E. coli* or coliforms, might function as indicator bacteria for EEC in the broiler processing line [31, 32].

In this review, we therefore provide an overview on data concerning intervention measures to quantitatively reduce* E. coli*, coliforms, or Enterobacteriaceae during processing of broiler chickens. We evaluated data from 73 original research papers and summarized the quantitative effects of the investigated interventions to reduce these bacteria at the different stages of the slaughter processing line (supplementary table (available here)). The studies comprise interventions to reduce/prevent the contamination of carcasses during processing as well as to remove the contamination that already occurred. There are also some data available concerning certain measures directly prior to the transport of broiler chickens to the slaughterhouse to prevent fecal material from the fattening farms entering into the processing line as well as those measures which refer to slaughterhouse equipment or cleaning and disinfection procedures.

## 2. Intervention Measures

### 2.1. Prior to Processing

Despite general biosafety and biosecurity measures as well as cleaning and disinfection strategies in the fattening period of broiler chickens, further preprocessing factors like chicken age, feed withdrawal, or water and feed additives might lead to a reduced introduction of Enterobacteriaceae, in particular* E. coli* and coliforms, into the slaughterhouses and therefore reduced cross-contamination during carcass processing (Table 1).

It was found that increased age (56 days vs. 42 days) of broiler chicken led to higher contamination of the broiler carcasses with* E. coli* strains or coliforms after chilling in chlorinated water [33]. However, detailed data earlier in the processing line were not available and, therefore, data might be biased. Further studies investigated the influence of duration of feed withdrawal. In general, feed withdrawal of 8 to 10 hours is used to reduce the fecal amount in the gastrointestinal tract of broiler chickens and therefore a reduced probability of fecal contamination during automated evisceration is assumed [34, 35]. However, in four different studies from three different countries, various outcomes were observed [33, 36–38]. Results showed both a reduction and an increment of the* E. coli* amount on the carcasses in the different samplings suggesting insufficient effectiveness of the feed withdrawal concerning the reduction of* E. coli*. Furthermore, the effect of the feed withdrawal time varied between samples at different stations in the slaughter processing line [37]. This led to the assumption that the processing or certain steps of processing, e.g., evisceration, have a higher impact on the contamination rate of broiler carcasses with* E. coli* than the duration time of the feed withdrawal. In addition to feed withdrawal times, certain feed and water additives were investigated to enhance a possible positive effect of the feed withdrawal concerning the reduction of particular pathogens (Table 1). Substances like specialized replacement finisher [37], the supplementation of feed with a glucose cocktail [39], chlorine additives [36], or magnesium sulfate [40] in drinking water, or the supplementation of tap water with an allostatic modulator [38] were investigated. These substances were applied to broiler chickens prior to feed withdrawal. Again, various outcomes concerning a reduction of* E. coli*, Enterobacteriaceae, or coliforms concentrations on broiler carcasses were observed. Glucose feed additives and chlorine compounds in drinking water showed reductions of Enterobacteriaceae or* E. coli* in chicken crops [36, 39]. However, an impact on the reduction of* E. coli* on the broiler carcasses during processing and concerning the prevention on cross-contamination seemed to be ambiguous and needs further investigations. The supplementation of drinking water with magnesium sulfate at different levels led to a reduction of up to 2 log10 values of the microbial cecum contents and coliform bacteria [40]. However, in these experiments neither the effect on* E. coli* counts nor EEC counts were investigated and therefore the application of magnesium sulfate needs to be further evaluated. Overall, the investigated measures do not provide a distinct strategy for the reduction of the introduction of* E. coli* into slaughterhouse facilities. However, positive effects of these measures on the reduction of EEC numbers on broiler carcasses were not yet published.

### 2.2. During Processing

The processing of broiler chickens consists of the following major steps: arrival at the slaughterhouse, stunning, bleeding, scalding, defeathering, evisceration, washing, chilling, and cutting/packaging. The summarized study data include intervention measures during general online slaughter procedures as well as experimental trials in pilot processing plants or under laboratory conditions for all processing steps except stunning and bleeding (Table 2). For both these steps, no data or studies could be identified.

#### 2.2.1. After Arrival

Visible contamination of broiler chickens (on feathers and skin) is of great importance concerning the introduction of the bacteria into the slaughter facilities [27, 28]; however, there are only few studies which investigated measures before the scalding of the broiler chickens (Table 2).

In two independent studies, experimental prescald equipment was developed to brush broiler chickens before scalding to reduce the visible contamination and to lower the amount of fecal material and bacteria which were further introduced into the scalding water [41, 42]. The entering of fecal material into the scalding water has an effect on the pH of the scalding water. It decreases due to dissociation of the ammonium urate, present in chicken feces, into uric acid and ammonium hydroxide [43] and thus influences heat resistance of* Campylobacter* and* Salmonella* [44, 45]. However, for* E. coli* the pH of the scalding water seems to be less important [46]. Both prescald brushing studies used slightly different techniques (whole surface vs. breast, vent, and neck areas) and reductions of up to 0.3 log10 CFU of* E. coli*, coliforms, and Enterobacteriaceae on carcasses, respectively, might be achievable. The partial brushing of the breast, vent, and neck was not tested as an online treatment and, therefore, the effect after scalding could not be examined [42].

A second important contamination source is internal fecal material, which could contaminate carcasses due to leakage from the cloacae or gastrointestinal disruptions. Therefore, Northcutt et al. investigated in their study the impact of a forced cloacal fecal expulsion prior to scalding to prevent these leakages during further carcass processing [47]. They found no differences in the bacterial load between prescald washed, prescald squeezed, and prescald washed and squeezed carcasses but a comparison to untreated carcasses was not done. Musgrove et al. investigated the contribution of a cloacal plugging prior to electrocution of broilers to reduce numbers of Enterobacteriaceae on broiler carcasses [48]. The closure of the cloacae seemed to prevent further fecal contamination during processing as they determined reductions of 0.53 log10 CFU/ml per carcass rinse. In experimental inoculation trials, Buhr et al. also used manual cloacal plugging and vent suturing to prevent fecal leakage [49]. They found a reduction of 1.2 log10 CFU/ml breast skin rinse* E. coli* compared to broiler carcasses with open vents. Nevertheless, cloacal plugging is labor-consuming and is not yet established as an online procedure. However, it points to the fact that fecal leakage is a major problem in contamination of broiler carcasses.

Even though there are only limited studies on preprocessing measures, there might be a great potential in new strategies for the quantitative reduction of the bacterial load on broiler chickens before their processing.

#### 2.2.2. Scalding

Scalding of carcasses is used to preliminary loose the feathers of broiler carcasses prior to the actual defeathering process. Two different systems of carcass scalding are established: immersion scalding in water bathes and stream/spray scalding. During immersion scalding, bacteria are removed by the effect of high temperature and a washing effect of the water bath. However, cross-contamination was shown during immersion scalding [50, 51]. During steam scalding, bacteria are only reduced via a temperature effect but cross-contamination is expected to be less likely [52]. In the course of the development of the slaughter processing, different immersion scalding conditions have been established. Hard scalding with water temperatures from 60 to 66°C and immersion time of 45 to 90 s were typically used in the US poultry industry whereas in Europe soft scalding at 51 to 54°C with immersion times for 120 to 210 s is preferred [53]. The scalding conditions are also linked to the preferences of consumers with regard to certain attributes of fresh chicken meat like color of meat and skin [54] and the form of offer, chilled or frozen. Early data from artificial contamination trials of chicken skin with an* E. coli* K12 strain indicate that scalding temperatures above 60°C (scalding time of 150 s) led to an increased reduction of this strain from the contaminated chicken skin [55]. They also concluded from their results that it seems to be difficult to totally remove the* E. coli* strain from the chicken skin as it is protected by polymers on the surface of the chicken skin. Mulder et al. found in their artificial contamination study that cross-contamination during scalding is very likely and that external contamination might be of greater importance than internal contamination [56]. Due to the need for enhanced hygiene measures to reduce microbiological contamination and consequently foodborne illnesses via poultry food products, further investigations were carried out to improve the effectiveness of the scalding process without leading to a reduced meat quality and meat appearance.

Later on, countercurrent immersion scalding and additional postscald hot water sprays have been introduced [57]. However, the use of a three-tank counterflow scalder does not lead to a microbiological improvement of the contamination level of broiler carcasses compared to a single-tank scalder [58] although there was a reduction of aerobic bacteria in the last scalding tank. Berrang et al. found that the scalding in a counterflow triple-tank scalder decreased the* E. coli* counts from 4.6 log10 CFU to 2.0 log10 CFU, but controls to other scalding techniques were not provided [59]. Further studies showed that variations in the pH of the scalding water [46] as well as technical developments of immersion scalders like triple-tank scalders [60], counterflow triple tanks [61], or an additional cold water scald [62] also had no distinct effect on the amount of* E. coli* or coliforms detected on the broiler carcasses. In contrast, the addition of copper sulfate-based sanitizers [63] or ammonium chloride substances [64] to the scalding water seemed to be of great advantage in reducing the amount of* E. coli* or coliforms on broiler carcasses. However, these substances are not approved for use in European slaughterhouses as there are no chemicals approved for use in the European Union (EU) [65]. Further variations in the scalding temperatures for immersion and spray scalders were not tested concerning their influence on the microbiological status of the carcasses. It also seems to be challenging to achieve an equal distributed scalding temperature at the different sites of the carcasses and to avoid a “cooked” skin appearance when using higher scalding temperatures [52].

Further scalding techniques like spray or vapor scalding have been tested as it was assumed that these methods would reduce water consumption and the amount of waste water [66] and might also reduce possible cross-contamination [52]. A prototype of a steam-hot-water-spray scalder was tested and showed a reduction of coliforms of approximately 0.5 log10 CFU/cm^2^ on carcass surface compared to a conventional scalder [67]. However, there were apparently no further data determined on the efficiency of these techniques to reduce the microbiological load on broiler carcasses.

As the scalding process of broiler carcasses was identified as a critical point for cross-contamination events with pathogenic foodborne bacteria [50, 51] as well as ESBL-producing Enterobacteriaceae [24, 68], it is necessary to improve the processing in the context of microbiological hygiene measures.

#### 2.2.3. Defeathering

The defeathering process aims at the complete removal of the feathers from the broiler carcasses while keeping the skin and carcass appearance according to consumer preference. The defeathering machine consists of banks with sets of motor driven discs with rubber plucking fingers. This process also turned out to be critical concerning microbiological contamination of broiler carcasses as during defeathering the pressure may be released to the carcasses which frequently leads to fecal leakage [51, 69–71]. It was recently shown that also cross-contamination with ESBL-producing Enterobacteriaceae can occur [24, 68]. Only few studies were conducted to investigate the impact of the defeathering process on microbiological contamination. Cason et al. found no significant difference in the numbers of* E. coli* of carcasses after mechanical defeathering for 30 s and 60 s, respectively, in a laboratory processing facility [72]. Allen et al. reported that the majority of feathers with attached bacteria were already removed in the first 10 sec of the defeathering process [70]. To possibly reduce the microbiological load on the carcasses directly after defeathering, hot water immersion scald and spray washer were investigated [41, 73, 74]. The studies found reductions between 0.2 log10 and 0.7 log10 of the* E. coli* load in the whole carcass rinses. However, limitations were alterations in meat quality and meat appearance due to high water temperatures and the use of chlorinated water which is not approved in the EU [65]. The effects of an application of acetic acid and hydrogen peroxide during defeathering on the microbiological quality were only tested for total aerobic plate counts but not for* E. coli* [75].

Overall, there are only few studies available/published, which dealt with microbiological investigations during defeathering of carcasses. Respective studies might not be conducted or the investigations are just not available to the public. It also seems that further development of defeathering technology has a greater focus on processing parameters like defeathering efficacy than on microbiological aspects.

#### 2.2.4. Evisceration

The aim of this processing step is to remove the total intestinal package. The whole evisceration process is highly automated and most challenging is the proper evisceration of highly variable sizes of broiler carcasses without leading to fecal leakage and gastrointestinal disruptions. There is limited information about the effectiveness and possible microbiological contamination due to the technologies used for every single evisceration step. Russel et al. did early comparisons between the Nu-Tech Evisceration System and a conventional Streamlined Inspection System (SIS). Evisceration with the Nu-Tech system leads to the separation of the visceral package from the carcass for inspection whereas with the SIS the package remains attached to the carcass. The Nu-Tech system showed better performance concerning the visible fecal contamination of the carcasses but no difference in the amount of* E. coli* in the investigated carcass rinses between both systems was observed [76].

Compliance with procedures to set and control equipment may be also associated with presence of fecal contamination [77]. This contamination occurs as a result of damage of the intestines due to heterogeneity of carcasses within and between flocks. Conventional equipment cannot be adjusted per carcass; however, evisceration employees can adjust it for a particular flock to minimize the fecal leakage. The observed association between compliance with procedures and occurrence of fecal contamination needs to be validated in intervention studies.

The structure of the skin of broiler chickens is assumed to play an important role in level of observable microbiological contamination due to the properties of the skin surface and the associated polymers which can protect bacteria from removal [55, 78]. Therefore, whether the removal of the skin prior to evisceration can lead to reduced contamination of the chicken meat was investigated. After manual evisceration, they found a reduction of 0.5 log10 CFU/carcass of* E. coli* and coliforms, respectively [79]. However, the possible reduction of contamination with Enterobacteriaceae by removing broiler chicken skin before evisceration was not tested as an online operation.

#### 2.2.5. Postevisceration Treatment

Leakage of fecal material and contamination with bacteria occur due to improper efficiency of the evisceration. Therefore, a high number of investigations were conducted to improve the removal of (visible) fecal contamination on broiler carcasses by improving the washing technologies as well as adding various substances to the washing water.

Early examinations on spray washing and inside-outside (I/O) washers revealed slight reductions of Enterobacteriaceae on broiler carcasses due to this application [80]. Further investigations of I/O washers also found only slight reductions of the* E. coli* and coliform count, respectively, even though chlorinated water was used for the washing process which is not approved in the EU [41, 65, 74, 80, 81]. Furthermore, it was shown that an I/O wash with a showering time of 5s to 6s does not completely remove visible fecal contamination and, therefore, is less effective in reducing* E. coli*, Enterobacteriaceae, or coliforms from contaminated broiler carcasses [25]. Berrang and Baily examined a post-I/O brush wash step and found an additional reduction of about 0.5 log10 CFU of* E. coli* and coliforms, respectively [41]. The trimming of visible fecal contamination with different water pressure resulted in a lower reduction of these bacteria [82] and the usage of high pressure spray with chlorinated water showed varying outcomes in the ability to reduce Enterobacteriaceae from broiler carcasses [83]. Studies also investigated the efficiency of water washes to reduce the numbers of* E. coli* or coliforms on broiler carcasses. Experiments were carried out in the lab or in experimental pilot processing plants and it turned out that using water with less than 2 ppm chlorine or potable water for the washing steps led to reductions between 0.3 and 1.3 log10 CFU of* E. coli*, coliforms, or Enterobacteriaceae [82, 84, 85].

Most of the interventions against* E. coli*, coliforms, or Enterobacteriaceae on broiler carcasses by different washing steps or technologies after the evisceration include the usage of chlorinated water (produced, e.g., by adding sodium hypochloride (SH), or by electrolyzing water containing dissolved sodium chloride) which is not approved in the EU [65]. Here, investigations in the lab or in processing plants showed better results concerning the reduction of* E. coli*, coliforms, and Enterobacteriaceae, respectively, than online investigations in the slaughterhouse [84–87]. Furthermore, a study concerning online postevisceration washes with chlorinated water resulted in higher bacterial contamination than without chlorinated water [74]. The same was found in a study concerning prechill washes [41] whereas chlorine dioxide (ClO_2_) spray wash seems to reduce* E. coli* and coliform counts about 0.4 log10 CFU, respectively [74]. Kemp et al. investigated the possible usage of acidified sodium chlorite (ASC) as an intervention against* E. coli* contamination on broiler carcasses [88, 89]. They conducted lab work experiments and combined online treatments and found reductions between 0.77 and 2.28 log10 CFU.

Further substances like trisodium phosphate (TSP), lauric acid (LA), myristic acid (MA), or potassium hydroxide (KOH) were also tested in further studies as candidates to potentially reduce* E. coli*, coliforms, or Enterobacteriaceae counts on broiler carcasses after the evisceration step. Outcomes vary between 0.33 and 2.07 log10 CFU reduction [74, 85, 90, 91]. Acetic acid (1.4 g/l and 2.8 g/l AA) and oleic acid as a 10% washing solution were also tested in lab experiments. They showed reductions of up to 0.93 log10 CFU of* E. coli* and 2.43 log10 CFU of Enterobacteriaceae, respectively, on poultry skin samples [92, 93]. For kosher chicken meat production, the usage of salt was evaluated after the evisceration step of the carcasses [94]. During their investigation, they found that kosher salt application can reduce the* E. coli* and coliform counts by 2.81 and 2.31 log10 CFU, respectively.

Most of the studies dealt with washing substances which are not approved in Europe [65] (Table 2). It turned out that the addition of chlorine compounds does not lead to a total removal of Enterobacteriaceae on broiler carcasses and also more natural substances might have a potential to reduce these bacteria on broiler carcasses during processing. However, most investigations were done as lab work or in pilot processing plants and the results need to be further evaluated.

#### 2.2.6. Chilling

The chilling process in general can lead to a reduction of the* E. coli* amount on broiler carcasses of up to 3.5 log10 values [95, 96]. However, broiler carcasses are still contaminated with* E. coli*, coliforms, or Enterobacteriaceae after chilling. This was also to be found for multidrug resistant* E. coli* like EEC [97–99].

Chilling of broiler carcasses is done via immersion chilling in a water tank, chilling in air, or air-spray chilling where carcasses are sprayed with water at several points in the chilling room. Furthermore, some slaughterhouses use a combination of immersion and air chilling having a water bath with cold water only in the beginning of the chilling room. The chilling method applied depends on the scalding temperature regime. If the scalding temperatures are very high, the epidermis is removed and the carcasses need to be kept wet through the process; otherwise, the appearance of the chicken skin/meat is affected. Therefore, chilling in a water bath is applied in combination with high temperature scalding.

The use of a water bath during chilling—like the scalding water bath—might also contribute to cross-contamination between carcasses but experimental studies on air chilling with or without chlorinated water sprays did not result in reductions of the amount of* E. coli* or coliforms on the broiler carcasses [100–105]. The use of steam or hot water in combination with rapid cooling, chilling, or freezing seems to reduce up to 2.83 log10 CFU of* E. coli* depending on the treatment time [106]. However, there are disadvantages in the skin appearance and skin color [106]. Freeze chilling of chicken meat for longer transportation was investigated with no negative effect on the meat appearance and found to possibly reduce up to 1 log10 CFU of Enterobacteriaceae on the chicken meat [107]. In contrast, an experimental trial on the survival of* E. coli* K12 during crust freezing resulted only in reductions 0.3 log10 CFU [108]. Allen et al. investigated (chlorinated) water sprays for carcass chilling but the effect in reducing coliforms from breast skin was not more than 0.62 log10 CFU even when using 250 ppm of chlorine [100].

Immersion chilling in tap water showed reductions of up to 1.1 log10 CFU of* E. coli* under laboratory conditions compared to unchilled carcasses [109]. Chilling in water with less than 2 ppm free chlorine residues reduced* E. coli* counts by 1.34 log10 CFU [110]. In the same study, after renewal of the chilling water after 8 h (instead of 16 h) the reduction was determined as 1.25 log10 CFU of* E. coli*. In most of the studies, the use of chlorine substances or solutions like acidified chlorine, sodium hypochlorite (SH), monochloramine (MON), or chlorine dioxide (ClO_2_) for the reduction of bacterial contamination on broiler carcasses was investigated [25, 33, 60, 74, 111–113]. On the one hand, reductions of up to 1.4 log10 CFU of* E. coli* were detected. On the other hand, also increments of the amount of* E. coli* (up to 0.2 log10 CFU) were found. This might be due to the sampling of different slaughterhouses and different sampling conditions/methods and laboratory work. In the study by Kameyada et al., they found that also the chilling temperature might have an influence on the reduction of* E. coli* counts on broiler carcasses [112]. To further reduce the bacterial contamination from the carcasses and additionally prevent cross-contamination, Dickens et al. investigated an herbal extract as additive in the chilling water [109]. In their laboratory, they determined reductions of 2 log10 CFU and 2.64 log10 CFU for* E. coli* and coliforms, respectively. Postchill washing or dipping steps in chlorinated water can lead to additional reductions between 0.2 and 1.74 log10 CFU of* E. coli* or coliforms on the broiler carcasses [74, 81, 87]. A prechill or postchill application of kosher salt to the carcasses was found to reduce* E. coli* counts by 1.39 log10 CFU and 1.77 log10 CFU, respectively [94].

The analysis of various published papers concerning the microbiologic profile of broiler carcasses after chilling highlights the need for harmonized methods and sampling procedures. It also shows that, in concordance to the postevisceration wash, the adding of chlorine compounds to the chilling water does not completely remove Enterobacteriaceae from the broiler carcasses assuming that methods for preventing contamination of broiler carcasses might be of greater importance.

### 2.3. Packaging

There are different technologies established to protect raw meat from recontamination and to prevent the growth of potential pathogenic bacteria [114]. For the preservation of chicken meat, investigations were conducted to improve the shelf life and to reduce bacterial contamination (Table 3). Various combinations of gaseous substances and concentrations were tested in modified atmosphere packaging (MAP) processes. Most common are combinations of the gases O_2_, CO_2_, and N_2_. Depending on the storage time investigated in the studies, most of the tested MAP gases showed reduced growth of* E. coli* or Enterobacteriaceae compared to the storage under air conditions [107, 115–117]. High portions of CO_2_ in the gaseous mixtures seemed to better reduce the growth of* E. coli* on chicken meat [116, 117]. However, none of the tested MAP processes led to a total reduction of* E. coli* counts on chicken meat. The shelf life of chicken meat is not only dependent on the amount of* E. coli* on the respective filets or chicken wings. Therefore, most of the investigations on MAP also concentrate on other bacteria like pseudomonads and other potential pathogenic Enterobacteriaceae as well as meat appearance and consumer preferences [54, 114, 118]. As alternatives to MAP, active packaging has been developed which leads to an interaction of the packaging material and the respective meat [119]. Furthermore, the packaging material can be incorporated with different (reactive) substances to increase the shelf life due to the interaction of material, substances, and meat. The substances allowed for use for active packaging are also strictly regulated in the EU [119]. The incorporation of 3% carvacrol or 3% cinnamaldehyde into wrapping films reduced the amount of* E. coli* O157:H7 on chicken breast samples of up to 6.8 and 5.2 log10 CFU, respectively, after storage time of 72 h at 23°C [120]. Using ovotransferrin or potassium sorbate showed reductions of more than 2 log10 CFU of* E. coli* only in combination with 5 mM EDTA whereas the reduction due to EDTA alone was higher than for both separate substances [121]. Besides the packaging technology, the decontamination of chicken meat with a water extract of sumac (WES) and 2% lactic acid (LA) was investigated in a broiler wing model [122]. Reductions up to 2 log10 CFU of coliforms were detected compared to a distilled water reference. Haughton et al. determined the decontamination of chicken meat using high-intensity pulsed light (HIPL) and found a reduction of up to 1.51 log10 CFU of* E. coli* on uncovered chicken skin [123]. However, the use of HIPL on packaged chicken meat/skin led to a lower reduction of the* E. coli* amount.

None of the investigated interventions was tested for their efficacy against EEC. However, it seems that the reduction or eradication of EEC needs to be done in previous steps in the processing of broiler chickens.

### 2.4. Equipment/Others

Despite direct intervention measures to reduce* E. coli*, coliforms, or Enterobacteriaceae on broiler carcasses also few investigations on the reduction of bacterial contaminants in the slaughterhouse environment were conducted (Table 4). These investigations include the general sanitary treatment in slaughterhouses and the disinfection of conveyor belts and transport crates. In the study of Kašková et al., no coliforms were detected on the sampled sites, except for the shackling hooks, after disinfection with quaternary ammonium compounds [124]. For the sanitizing of stainless steel sodium hypochloride (SH) and peracetic acid were tested under laboratory conditions [125]. From the results, the authors did not recommend peracetic acid as a sanitizing agent for slaughterhouse equipment. Cleaning and disinfection of conveyor belts and transport crates are critical concerning cross-contamination. Hot water treatment does not result in significant reduction of Enterobacteriaceae whereas washing, soaking, and the additional use of disinfectants or detergents are more effective [126–128]. Ultrasonic treatment of conveyor belts was more effective in combination with water temperatures around 60°C [127]. These studies show that the disinfection of the slaughterhouse equipment or transport crates is a critical process and needs to be done accurately to avoid cross-contamination and further spread of the bacteria.

For the decontamination of chilling wash water Rowan et al. tested a pulsed-plasma gas discharge system [129]. They found a reduction of approx. 8 log10 CFU of* E. coli* NCTC9001 after a treatment time of 18 sec. UV irradiation and LED light were further methods tested for the decontamination of stainless steel and chicken skin [130, 131]. UV irradiation showed better results concerning the reduction of* E. coli* on stainless steel (up to 5.34 log10 CFU) than on chicken skin (up to 1.28 log10 CFU) [130]. This is assumed to be due to the rough surface of the chicken skin and the feather follicles which protect bacteria from the UV light. The LED array treatment did not exceed 1 log10 CFU reduction on stainless steel and chicken skin, respectively [131]. Furthermore, the treatment period ranged between 10 and 20 min which might be problematic for an installation as online treatment.

There is a high diversity in methods tested for the inactivation or reduction of Enterobacteriaceae or* E. coli* counts in slaughterhouses or slaughterhouse equipment. However, most of the methods were not tested for their potential to also reduce contamination on chicken carcasses or as an online intervention in a slaughterhouse.

## 3. Summary and Conclusion

ESBL-producing Enterobacteriaceae are frequently detected in broiler chickens and chicken meat. Due to high prevalence of these usually multidrug resistant bacteria, an impact on human health is assumed [4, 5]. It was recently reported that reduced exposure to humans also led to a reduction in the prevalence in humans [132]. The transmission along the broiler production chain and certain cross-contamination events have been described by various authors [12–15]. Furthermore, it has been reported that wastewater from processing facilities contributes to the spread of multidrug resistant bacteria into the environment [133–136]. Therefore, interventions are needed to reduce or even eradicate these ESBL-producing Enterobacteriaceae from the broiler production.

Until now certain interventions were investigated against* Campylobacter* sp. and/or* Salmonella* sp. but specific interventions against EEC were not evaluated. We, therefore, summarized data from various studies which also investigated Enterobacteriaceae counts with* E. coli* and coliforms in particular as they might function as indicator bacteria for EEC in the broiler processing line [31, 32].

Overall, we found 73 studies providing data on the quantitative reduction of* E. coli*, coliforms, or Enterobacteriaceae along the different steps of the broiler processing line (supplementary table). Reductions were measured up to 3 log10 CFU on chicken skin or broiler carcasses; however, none of the methods led to total eradication of those bacteria. A variety of investigated measures provided only reductions below 1 log10 CFU or even caused an increase in the respective bacterial counts indicating an insufficient effect against* E. coli*, coliforms, or Enterobacteriaceae contamination of broiler carcasses and, therefore, an effect on EEC is questionable. In addition, it seems that experimental intervention trials provide better results than measures implemented as online treatment. Also, the effect of measures dependent on the contamination level of broiler carcasses is not well investigated. The application of simultaneous or parallel interventions might have an additive effect; however, respective studies for most of the interventions are missing.

We found studies comprising interventions to prevent fecal material from the fattening farms entering into the processing line of the slaughterhouse (measures prior to processing and after arrival), to reduce/prevent the contamination of carcasses during processing (scalding, evisceration) as well as to remove contamination that already occurred (postevisceration treatment, chilling, packaging, and equipment/others). It was already reported that the structure of the chicken skin plays an important role in attachment of bacteria and that firmly attached bacteria during plucking are more difficult to remove [51, 55, 137]. This might suggest that there is a need for more measures that prevent contamination from occurring.

Twenty-one measures dealt with the application of various chlorine substances which are not approved in the EU [65]. The overall reduction of* E. coli*, coliforms, or Enterobacteriaceae by these substances was less than 2.3 log10 CFU, indicating that chlorine does not remove* E. coli*, coliforms, or Enterobacteriaceae from broiler carcasses to a preferable amount. Again, taking into account the important issue of skin structure and the bacterial attachment, it could be concluded that there are general limitations to the effectiveness of decontaminating chicken carcasses. It could be of interest to develop more interventions preventing the introduction or the recontamination with Enterobacteriaceae especially under the current EU regulations where decontamination is not approved. From our collection, 42 studies were already published before 2008 and, therefore, it is very likely that some of these measures are already installed in slaughterhouses. Furthermore, 58% of the studies (n=42) were conducted in the USA and only 21% in European countries (n=15) which highlights the need for further investigations in the EU due to differences in the respective guidelines and European decrees.

In the study of Pacholewicz et al., they found an overall reduction of EEC on broiler carcasses during processing [29]. However, they also detected differences in the reduction during processing of* E. coli* and EEC populations in one slaughterhouse. Here, EEC were less reduced than the numbers of total* E. coli* after defeathering and after evisceration, respectively. Furthermore, the effect of a respective processing step on the reduction of EEC additionally illustrated differences between two slaughterhouses [29]. This indicates that also slaughterhouses specific intervention measures might be needed against the dissemination of EEC. In addition, effective measures might depend on the current equipment, procedures, and technologies used in every single slaughterhouse, which need to be further evaluated.

Nevertheless, there is a need to eliminate as much as possible at various stages of processing (through preventing fecal contamination from entering a particular step) and maintaining and/or further reducing the amount of bacteria achieved at a respective processing step. EEC do not only spread in the broiler production line but can also be spread into the environment via wastewater and, therefore, contribute to the transmission of antibiotic resistance factors. Intervention measures are needed to prevent the spread and cross-contamination of these resistant bacteria during slaughter and finally the transmission into the environment and the households via contaminated chicken meat.

## Figures and Tables

**Table 1:** Overview of treatments/intervention measures prior to processing.

**Measures prior to processing**	
Age of broilers before slaughter	42 d, 49 d, 56 d
Feed withdrawal	4 h to 16 h
Replacement finisher	
Feed additives	Glucose
Water additives	Chlorine, MgSO_4_, allostatic modulator

**Table 2:** Overview of treatments/intervention measures during processing.

**After arrival**	**Scalding**	**Defeathering**	**Evisceration**	**Postevisceration treatment**	**Chilling**
Prescald brushing	Temperature of scalding water	Defeathering time	Nu-Tech Evisceration System	Spray washes	Air chilling
Forced cloacal fecal expulsion	Steam scalding	Postdefeathering scald	Skin removal prior to evisceration	Inside-outside (I/O) washes	Steam treatment (hot water)
Cloacal plugging	Countercurrent/counterflow scalding	New York dressed (NYD) carcass spray washes		Post-I/O brush wash	Rapid cooling/freeze chilling of carcasses/crust freezing
	pH of scalding water			Trimming of visible fecal contamination	Chilling with water sprays/chlorinated water sprays
	Triple/multiple immersion scalding tanks			Trimming/high pressure sprays	Chilling in tap water
	Postscald cold water treatment			Water washes (potable water)	Immersion chilling in chlorinated water
	Water additives (commercial sanitizer, Timsen®)			Water washes/spray washes using electrolyzed water/acidified electrolyzed water supplemented with sodium hypochloride (SH)/chlorine dioxide (ClO_2_)/acidified sodium chlorite (ASC)	Chilling in water with sodium hypochloride (SH)/monochloramine (MON)/chlorine (Cl_2_)/acidified chlorine (ClO_2_-Cl_2_)
				Water additives like trisodium phosphate (TSP), tripotassium phosphate (TTP), lauric acid (LA), myristic acid (MA), potassium hydroxide (KOH), acetic acid (AA), oleic acid	Chilling water with 2% Protecta II (herbal extract on an NaCl carrier)
				Kosher salt application	Postchill dips/washes
					Postchill spray wash with electrolyzed water (EO)
					Kosher salt application

**Table 3:** Overview of packaging treatments/intervention.

**Packaging**	**Substances**
Modified atmosphere packaging (MAP)	Various combinations of O_2_, CO_2_, and N_2_
Decontamination	Water extract of sumac, lauric acid (LA), high-intensity pulsed light
Active packaging	Carvacrol, cinnamaldehyde, ovotransferrin, potassium sorbate

**Table 4:** Overview of treatments of equipment/slaughterhouse environment.

**Equipment**
Conveyor treatment
Disinfectants (peracetic acid and quaternary ammonium, sodium hypochloride (SH), peracetic acid)
Transport crate treatment
LEDs/UV light

## Supplementary Materials

Supplementary Materials Supplementary table: published study data on quantitative reductions of* E. coli*, coliforms, or Enterobacteriaceae in the broiler processing line (negative reductions meaning an increase in the bacterial load are marked in red).
