# Supplementary material for: Reviewing Interventions against Enterobacteriaceae in Broiler Processing: Using Old Techniques for Meeting the New Challenges of ESBL E. coli?
Source: Biomed Res Int. 2018 Oct 23;2018:7309346. doi: 10.1155/2018/7309346 (PMC6218796; doi:10.1155/2018/7309346)
Supplement: Supplementary Materials — Supplementary table: published study data on quantitative reductions of E. coli, coliforms, or Enterobacteriaceae in the broiler processing line (negative reductions meaning an increase in the bacterial load are marked in red). [file 7309346.f1.docx]

Supplementary Table: Published study data on quantitative reductions of *E. coli*, Coliforms or Enterobacteriaceae in the broiler processing line (negative reductions meaning an increase in the bacterial load are marked in red)

| Intervention | Efficiency of reduction on carcasses | Comments | Reference (Country) |
| --- | --- | --- | --- |
| **Prior to Processing** |  |  |  |
| Age (42 days) | 0.6 log10 CFU/carcass *E. coli* (compared to 56 days of age)  0.3 log10 CFU/carcass *E. coli* (compared to 49 days of age)  0.4 log10 CFU/carcass Coliforms (compared to 56 days of age)  0.3 log10 CFU/carcass Coliforms (compared to 49 days of age) | - cotton plugs were inserted into the cloaca during bleeding - CFU counting after chilling in chlorinated water (not approved in the EU) | ([Northcutt et al., 2003a](#_ENREF_48))(USA) |
| Feed withdrawal (12h) | 0.2 log10 CFU *E. coli* (compared to 0h)  0.1 log10 CFU Coliforms (compared to 0h) | - cotton plugs were inserted into the cloaca during bleeding - CFU counting after chilling in chlorinated water (not approved in the EU) - Inoculation trial with Salmonella | ([Northcutt et al., 2003a](#_ENREF_48))(USA) |
| Feed withdrawal (12h) | 0.83 log10 CFU/g cecal content *E. coli* (compared to 0h)  -3.27 log10 CFU/ml of crop swab solution *E. coli* (compared to 0h) |  | ([Barreiro et al., 2012](#_ENREF_7))(BRA) |
| Feed withdrawal (16h) | ~0.38 log10 CFU/ml carcass rinse Coliforms (compared to 10h) | - Experimental farm - Sampling after manual evisceration | ([Rubio-Garcia et al., 2015](#_ENREF_59))(MEX) |
| Feed withdrawal | 0.4/0.5 log10 CFU/carcass *E. coli* (4h compared to 0h)  1.0/-0.5 log10 CFU/carcass *E. coli* (8h compared to 0h)  0.0/-1.1 log10 CFU/carcass *E. coli* (12h compared to 0h)  0.3/0.8 log10 CFU/carcass Coliforms (4h compared to 0h)  0.9/-0.5 log10 CFU/carcass Coliforms (8h compared to 0h)  -0.2/-1.0 log10 CFU/carcass Coliforms (12h compared to 0h) | - CFU counting of NYD carcasses/after evisceration | ([Northcutt et al., 2003b](#_ENREF_49))(USA) |
| Replacement finisher (8h before feed withdrawal) | 0.1/0.1 log10 CFU/carcass *E. coli* (4h compared to 0h)  0.7/-0.1 log10 CFU/carcass *E. coli* (8h compared to 0h)  0.2/-0.2 log10 CFU/carcass *E. coli* (12h compared to 0h)  0.0/0.4 log10 CFU/carcass Coliforms (4h compared to 0h)  0.5/0.0 log10 CFU/carcass Coliforms (8h compared to 0h)  0.0/-0.1 log10 CFU/carcass Coliforms (12h compared to 0h) | - CFU counting of NYD carcasses/after evisceration | ([Northcutt et al., 2003b](#_ENREF_49))(USA) |
| Glucose cocktail during feed withdrawal | 7.5% glucose  0.49 to 1.17 log10 CFU/g crop Enterobacteriaceae  15% glucose  0.54 log10 CFU/g crop Enterobacteriaceae | - Inoculation trial with Salmonella | ([Hinton et al., 2000](#_ENREF_30))(USA) |
| Chlorine drinking water during feed withdrawal (12h) | 0.02 log10 CFU/g cecal content *E. coli*  3.84 log10 CFU/ml of crop swab solution *E. coli* | - Chlorine not approved in the EU | ([Barreiro et al., 2012](#_ENREF_7))(BRA) |
| MgS04 via drinking water (24 h before processing) | 5.5g/L compared to untreated controls  ~1.8 log10 CFU/g intestine contents Coliforms  ~0.3 log10 CFU/g caecum contents Coliforms  ~1.0 log10 CFU/cm2 breast skin Coliforms | - levels of 0, 2.5, 4.0, 5.5, 7.0, and 8.5 g/L tested - swab of 12 cm2 breast skin before evisceration | ([Stanley et al., 1992](#_ENREF_69))(USA) |
| Allostatic modulator in tap drinking water (48h before shipment) | 10h feed withdrawal  ~0.29 log10 CFU/ml carcass rinse Coliforms  16h feed withdrawal  ~0.92 log10 CFU/ml carcass rinse Coliforms | - combined use of 50 mg AA, 62.5 mg ASA, and 251 μEq Na+,K+,and Cl− per L drinking water - Experimental farm - Sampling after manual evisceration | ([Rubio-Garcia et al., 2015](#_ENREF_59))(MEX) |
|  |  |  |  |
| **After arrival** |  |  |  |
| Pre-scald brushing  (whole surface) | 0.19 log10 CFU/ml carcass rinse *E. coli*  0.17 log10 CFU/ml carcass rinse Coliforms | - use of chlorinated water? | ([Berrang and Bailey, 2009](#_ENREF_9))(USA) |
| Pre-scald brushing  (breast, vent, neck) | 0.3 log10 CFU/carcass *E. coli*  0.3 log10 CFU/carcass Enterobacteriaceae | - Prototype intervention apparatus - not established on-line | ([Pacholewicz et al., 2016](#_ENREF_53))(NL) |
| Forced Cloacal Fecal Expulsion (Squeeze + Wash) | No differences between wash, squeeze and squeeze + wash | - No untreated controls - Genetically featherless broiler | ([Northcutt et al., 2008](#_ENREF_50))(USA) |
| Cloacal plugging | 0.53 log10 CFU/ml carcass rinse Enterobacteriaceae | - rinse samples from New York dressed broilers - not established on-line | ([Musgrove et al., 1997](#_ENREF_46))(USA) |
| Cloacal plugging | Feathered  1.2 log10 CFU/ml breast skin rinse *E. coli*  1.4 log10 CFU/ ml breast skin rinse Coliforms  Featherless  2.1 log10 CFU/ml breast skin rinse *E. coli*  2.3 log10 CFU/ ml breast skin rinse Coliforms | - Manual cloacal plugging and vent suturing - Sampling after scalding and defeathering of feathered and featherless broiler - Scalding in chlorinated water? | ([Buhr et al., 2003](#_ENREF_15))(USA) |
|  |  |  |  |
| **Scalding** |  |  |  |
| Heat destruction (after 2.5 min scalding) | 59.5°C  ~1.5 log10 CFU/g skin *E. coli* K12  62.5°C  ~3 log10 CFU/g skin *E. coli* K12  65.8°C  ~4 log10 CFU/g skin *E. coli* K12 | - Lab work, 3g skin, 2.5 min scalding - Artificial contamination trials | ([Notermans and Kampelmacher, 1975](#_ENREF_51))(NL) |
| Steam scalder | ~0.1 to 0.8 log10 CFU/cm² carcass surface coliforms (compared to conventional hot-water scalder) | - Prototype steam scalder - no significant differences after defeathering and chilling - data given only as diagrams | ([Patrick et al., 1972](#_ENREF_54))(USA) |
| Countercurrent scald+  Post scald spray | Preevisceration/prechill/postchill  2.09/1.61/0.89 log10 CFU/carcass *E. coli*  2.70/2.25/1.56 log10 CFU/carcass Enterobacteriaceae  Counter-current scald+spray wash  2.17/1.46/0.87 log10 CFU/carcass *E. coli*  3.07/2.29/2.32 log10 CFU/carcass Enterobacteriaceae | - No pre-scald/post-scald sample data for determination of efficiency of the scalding treatment | ([James et al., 1992](#_ENREF_35))(USA) |
| Counterflow triple-tank scalder | After scalding  2.6 log10 CFU/ml carcass rinse *E. coli*  2.7 log10 CFU/ml carcass rinse Coliforms  -1.5 log10 CFU/ml respiratory tract rinse *E. coli*  -1.8 log10 CFU/ml respiratory tract rinse Coliforms | - first tank for 1 min and 50 s, in the second tank for 57 s, and in the third tank for 45 s - no controls/comparisons included | ([Berrang et al., 2003](#_ENREF_12))(USA) |
| High pH scald  (mean pH 9.89) | No reductions on *E. coli* counts on carcasses compared to control pH (mean pH 6.88) |  | ([Berrang et al., 2011](#_ENREF_14))(USA) |
| Triple-tank scalder | -0.37 to 0.68 log10 CFU/carcass *E. coli* (compared to single-tank scald)  -0.41 to 0.69 log10 CFU/carcass Coliforms (compared to single-tank scald) | - Feathered and featherless broiler, sampling after chilling with water or chlorine - Different sampling methods in the trials | ([Buhr et al., 2005a](#_ENREF_16))(USA) |
| Counterflow multiple-tank scalder | No reductions of CFU on carcasses after defeathering | Scalding water  Tank 1: 3.2 log10 CFU/ml *E. coli*  Tank 2: 1.5 log10 CFU/ml *E. coli*  Tank 3: 0.8 log10 CFU/ml *E. coli* | ([Cason et al., 2000](#_ENREF_22))(USA) |
| Cold water scald (multiple-tank scalder) | No reductions of CFU on carcasses after defeathering | - No data for scalding water | ([Cason et al., 2001](#_ENREF_20))(USA) |
| Commercial sanitizer in scalding water | Only scalding  3.05 log10 CFU/carcass *E. coli*  Scalding and Post-Defeathering dip  1.21 log10 CFU/carcass *E. coli* | - Acidic, Copper Sulfate-Based Commercial Sanitizer (not approved in the EU) - Scalding and Post-Defeathering dip leads to lower reduction as only scalding | ([Russell, 2008](#_ENREF_60))(USA) |
| Timsen® (n-alkyl dimethyl benzyl ammonium chloride-40%) in scalding water | ~2.7 to 4 log10 CFU/g carcass rinse Coliforms | - 200ppm chlorine (not approved in the EU) | ([Lansini et al., 2017](#_ENREF_43))(BRA) |
|  |  |  |  |
| **Defeathering** |  |  |  |
| Defeathering time (60s) | 0.0/0.1 log10 CFU/ml carcass rinse *E. coli* (compared to 30s) | - Genetically feathered/featherless broiler siblings | ([Cason et al., 2004b](#_ENREF_21))(USA) |
| Post-defeathering hot water scald treatment | Immersion (28 s at 60 ± 1°C):  0.5 log10 CFU/carcass *E. coli*  0.5 log10 CFU/carcass Coliforms  Spray (20 s at 70 ± 2°C):  0.6 log10 CFU/carcass *E. coli*  0.3 log10 CFU/carcass Coliforms | - “No significant reductions under treatment conditions, which do not alter meat quality […].” - Pilot processing plant | ([Berrang et al., 2000](#_ENREF_11))(USA) |
| NYD spray wash | 0.56 log10 CFU/ml carcass rinse *E. coli*  0.64 log10 CFU/ml carcass rinse Coliforms | - Chlorinated water (not approved in the EU) | ([Berrang and Bailey, 2009](#_ENREF_9))(USA) |
| NYD spray wash | 0.2 log10 CFU/ml carcass rinse *E. coli*  0.2 log10 CFU/ml carcass rinse Coliforms | - Chlorinated water (not approved in the EU) | ([Stopforth et al., 2007](#_ENREF_70))(USA) |
|  |  |  |  |
| **Evisceration** |  |  |  |
| Nu-Tech Evisceration System | -0.2 to 0.2 log10 CFU/ml carcass rinse *E. coli* (compared to Conventional Streamlined Inspection System)  -0.2 to 0.1 log10 CFU/ml carcass rinse Coliforms (compared to Conventional Streamlined Inspection System) | - Stork Gamco Nu-Tech Evisceration System: separate presentation of the visceral package for inspection after removal from the carcass - conventional streamlined inspection system: viscera remains attached to the carcass during inspection - reduced visible fecal contamination with Nu-Tech | ([Russell and Walker, 1997](#_ENREF_62))(USA) |
| Skin removal prior to evisceration | 0.5 log10 CFU/carcass *E. coli*  0.5 log10 CFU/carcass Coliforms | - Evisceration by hand - Additional sponge sampling (external)   1.4 log10 CFU/sample *E. coli*  2.0 log10 CFU/sample Coliforms | ([Berrang et al., 2002](#_ENREF_10))(USA) |
|  |  |  |  |
| **Post-Evisceration treatment** |  |  |  |
| Spray wash | ~0.51 log10 CFU/ml carcass rinse Enterobacteriaceae | - Data from 4 slaughterhouses | ([Mulder and Bolder, 1981](#_ENREF_45))(NL) |
| I/O wash | 0.10 log10 CFU/ml carcass rinse *E. coli*  0.02 log10 CFU/ml carcass rinse Coliforms | - Chlorinated water (not approved in the EU) | ([Berrang and Bailey, 2009](#_ENREF_9))(USA) |
| I/O wash | ~0.26 log10 CFU/ml carcass rinse Enterobacteriaceae | - Data from 9 slaughterhouses | ([Mulder and Bolder, 1981](#_ENREF_45))(NL) |
| I/O wash | 1.06 log10 CFU/ml carcass rinse *E. coli* (2days)  -0.04 log10 CFU/ml carcass rinse *E. coli* (4days) | - Chlorinated water (not approved in the EU) | ([Oyarzabal et al., 2004](#_ENREF_52))(USA) |
| I/O wash | 0.3 log10 CFU/ml carcass rinse *E. coli*  0.3 log10 CFU/ml carcass rinse Coliforms | - Chlorinated water (not approved in the EU) | ([Stopforth et al., 2007](#_ENREF_70))(USA) |
| I/O wash | With visible fecal contamination  0.10 log10 CFU/ml carcass rinse *E. coli*  0.09 log10 CFU/ml carcass rinse Enterobacteriaceae  -0.02 log10 CFU/ml carcass rinse Coliforms  Without visible fecal contamination  0.23 log10 CFU/ml carcass rinse *E. coli*  0.36 log10 CFU/ml carcass rinse Enterobacteriaceae  0.18 log10 CFU/ml carcass rinse Coliforms | - 5-6s I/O showering time | ([Jimenez et al., 2003](#_ENREF_37))(ARG) |
| Post-I/O brush wash | 0.52 log10 CFU/ml carcass rinse *E. coli*  0.50 log10 CFU/ml carcass rinse Coliforms | - Chlorinated water (not approved in the EU) | ([Berrang and Bailey, 2009](#_ENREF_9))(USA) |
| Trimming of visible fecal contamination | 0.18 log10 CFU/g carcass *E. coli*  0.09 log10 CFU/g carcass Coliforms | - 490 to 588 kPa of pressure - Chlorinated water??? | ([Giombelli and Gloria, 2014](#_ENREF_25))(BRA) |
| Trimming+High pressure spray | -1.22 to 0.73 log10 CFU/g carcass *E. coli* | - 0.5 to 2.0 ppm of chlorine with 10 kg/cm2 of pressure - Chlorinated water (not approved in the EU) | ([Giombelli et al., 2015](#_ENREF_26))(BRA) |
| Washing in water | 0.4 to 1.3 log10 CFU/carcass *E. coli*  0.5 to 1.3 log10 CFU/carcass Coliforms | - Lab Work, fixed volume (400 mL) of water for a fixed time (1 min) in a plastic bag | ([Bartenfeld et al., 2014](#_ENREF_8))(USA) |
| Washing in water | Without fecal contamination  0.99 log10 CFU/g carcass *E. coli*  0.84 log10 CFU/g carcass Coliforms  With visible fecal contamination  1.04 log10 CFU/g carcass *E. coli*  0.92 log10 CFU/g carcass Coliforms | - less than 2.0 ppm of chlorine in the water (as determined by the Brazilian legislation) | ([Giombelli and Gloria, 2014](#_ENREF_25))(BRA) |
| Water spray wash | 5 sec spray  0.39 log10 MPN/cm2 Coliforms (compared to untreated control)  15 sec spray  0.42 log10 MPN/cm2 Coliforms (compared to untreated control) | - snow-hill yellow-feather breed - 105 days of age | ([Wang et al., 2018](#_ENREF_71))(CHN) |
| Final wash in potable water | 0.37 log10 CFU/g neck skin *E. coli*  0.31 log10 CFU/g neck skin Enterobacteriaceae | - “after defeathering” as control - Offline treatment dip | ([Whyte et al., 2001](#_ENREF_72))(IRE) |
| Washing in sodium hypochloride (SH) | 1.3 to 1.4 log10 CFU/carcass *E. coli*  1.3 to 1.4 log10 CFU/carcass Coliforms | - Lab Work, fixed volume (400 mL) of chlorinated (500 mg/kg) water for a fixed time (1 min) in a plastic bag | ([Bartenfeld et al., 2014](#_ENREF_8))(USA) |
| Washing in sodium hypochloride (SH) | 1.5 log10 CFU/ml carcass rinse *E. coli* | - pilot processing plant - inoculated cecal material | ([Northcutt et al., 2007](#_ENREF_47))(USA) |
| Spray wash with electrolyzed water (EO) with chlorine | 5 sec spray  0.34 log10 MPN/cm2 Coliforms (60 mg/L compared to water)  0.41 log10 MPN/cm2 Coliforms (30 mg/L compared to water)  15 sec spray  0.53 log10 MPN/cm2 Coliforms (60 mg/L compared to water)  0.60 log10 MPN/cm2 Coliforms (30 mg/L compared to water) | - snow-hill yellow-feather breed - 105 days of age | ([Wang et al., 2018](#_ENREF_71))(CHN) |
| Washing in acidified electrolyzed water (EO) with sodium hypochloride (SH) | 1.7 log10 CFU/ml carcass rinse *E. coli* | - pilot processing plant - inoculated cecal material | ([Northcutt et al., 2007](#_ENREF_47))(USA) |
| Final chlorine wash (sodium hypochloride, SH) | 1-2ppm SH  -0.18 log10 CFU/g neck skin *E. coli*  -0.09 log10 CFU/g neck skin Enterobacteriaceae  25ppm SH  0.12 log10 CFU/g neck skin *E. coli*  0.21 log10 CFU/g neck skin Enterobacteriaceae | - “after defeathering” as control - Chlorinated water (not approved in the EU) - “The broiler processing plant used in this study was located in Ireland […]. A separate evisceration water supply was used in the plant that normally contained a 25-ppm total residual chlorine concentration in the final carcass-washing system.” | ([Whyte et al., 2001](#_ENREF_72))(IRE) |
| Post-evisceration wash | -0.2 log10 CFU/ml carcass rinse *E. coli*  -0.3 log10 CFU/ml carcass rinse Coliforms | - Chlorinated water (not approved in the EU) | ([Stopforth et al., 2007](#_ENREF_70))(USA) |
| Pre-chill spray wash | -0.47 log10 CFU/ml carcass rinse *E. coli*  -0.55 log10 CFU/ml carcass rinse Coliforms | - Chlorinated water (not approved in the EU) | ([Berrang and Bailey, 2009](#_ENREF_9))(USA) |
| Pre-chill chlorine dioxide (ClO2) spray wash | 0.4 log10 CFU/ml carcass rinse *E. coli*  0.4 log10 CFU/ml carcass rinse Coliforms | - Chlorinated water (not approved in the EU) | ([Stopforth et al., 2007](#_ENREF_70))(USA) |
| Pre-chill I/O spray wash in acidified sodium chlorite (ASC) | Combined I/O wash and ASC treatment  0.77 log10 CFU/ml carcass rinse *E. coli* | - Lab work (I/O wash by hand) - Chlorinated water (not approved in the EU) | ([Kemp et al., 2000](#_ENREF_41))(USA) |
| Pre-chill I/O dip in acidified sodium chlorite (ASC) | Combined I/O wash and ASC treatment  1.23 log10 CFU/ml carcass rinse *E. coli* | - Lab work (I/O wash by hand) - Chlorinated water (not approved in the EU) | ([Kemp et al., 2000](#_ENREF_41))(USA) |
| Pre-chill I/O spray wash in acidified sodium chlorite (ASC) | Combined I/O wash and ASC online system  2.28 log10 CFU/ml carcass rinse *E. coli* | - Chlorinated water (not approved in the EU) | ([Kemp et al., 2001](#_ENREF_40))(USA) |
| Final wash in trisodium phosphate (10% TSP) | 1.95 log10 CFU/g neck skin *E. coli*  2.17 log10 CFU/g neck skin Enterobacteriaceae | - “after defeathering” as control - Offline treatment dip | ([Whyte et al., 2001](#_ENREF_72))(IRE) |
| Spray wash in trisodium phosphate (8-12% TSP) | 0.7 log10 CFU/ml carcass rinse *E. coli*  0.8 log10 CFU/ml carcass rinse Coliforms |  | ([Stopforth et al., 2007](#_ENREF_70))(USA) |
| Washing in tripotassium phosphate (TPP), lauric acid (LA), myristic acid (MA) | 1% TPP and 0.5% MA  0.50 log10 CFU/ml rinse *E. coli*  1% TPP and 0.5% LA  1.02 log10 CFU/ml rinse *E. coli*  2% TPP and 1.0% MA  0.58 log10 CFU/ml rinse *E. coli*  2% TPP and 1.0% LA  1.07 log10 CFU/ml rinse *E. coli*  3% TPP and 1.5% MA  0.35 log10 CFU/ml rinse *E. coli*  3% TPP and 1.5% LA   - 1. log10 CFU/ml rinse *E. coli*   4% TPP and 2.0% MA  0.2 log10 CFU/ml rinse *E. coli* | - Lab work, 5g neck skin | ([Hinton and Ingram, 2005](#_ENREF_31))(USA) |
| Washing in potassium hydroxide – lauric acid | 0.10% KOH  2.37 log10 CFU/ml *E. coli* PO1 (compared to pepton water)  0.10% KOH + 0.025% LA  4.52 log10 CFU/ml *E. coli* PO1 (compared to pepton water) | - Lab work - Growth reduction in Medium | ([Hinton and Ingram, 2006](#_ENREF_32))(USA) |
| Washing in potassium hydroxide – lauric acid (1% KOH + 2% LA) | 0.33 to 0.95 log10 CFU/ml carcass rinse *E. coli* (compared to distilled water) | - Lab work | ([Hinton et al., 2007](#_ENREF_33))(USA) |
| Washing in acetic acid | Water: 0.05-0.68 log10 CFU/skin sample *E. coli*  1.4 g/l AA: 0.05-0.93 log10 CFU/skin sample *E. coli*  2.8 g/l AA: 0.59-0.75 log10 CFU/skin sample *E. coli* | - Lab work, 25g skin | ([Jimenez et al., 2005](#_ENREF_36))(ARG) |
| Washing in oleic acid (10%) | 1.84 (one wash) to 2.43 log10 CFU/ml rinse Enterobacteriaceae (two washes) | - Lab work, 50g skin | ([Hinton and Ingram, 2000](#_ENREF_29))(USA) |
| Salt application | 2.81 log10 CFU/ml carcass rinse *E. coli* (compared to pre-chilled control)  2.31 log10 CFU/ml carcass rinse Coliforms (compared to pre-chilled control) | - Kosher salt: larger grain size compared with regular table salt (no dissolving when coating meat) - Lab work | ([Shin et al.](#_ENREF_65))(USA) |
|  |  |  |  |
| **Chilling** |  |  |  |
| Air chilling | No reduction in Coliforms on breast skin  1.01 log10 CFU/body cavity Coliforms | - Experimental rig | ([Allen et al., 2000a](#_ENREF_2))(UK) |
| Air chilling | -0.21 to -1.0 log10 CFU/body cavity Coliforms (compared to chilling in chlorinated water) | - Air with and without chlorinated water sprays - Chlorinated water (not approved in the EU) | ([Allen et al., 2000b](#_ENREF_4))(UK) |
| Air-chilling | -0.92 log10 CFU/cm2 skin *E. coli* (compared to water chilling)  -0.92 log10 CFU/cm2 skin Coliforms (compared to water chilling) | - chilling in water containing chlorine dioxide (not approved in the EU) | ([Barbut et al., 2009](#_ENREF_6))(CAN) |
| Air chilling  (150 min @ 1°C) | -0.56 log10 CFU/ml carcass rinse *E. coli* (compared to immersions chill in chlorinated water)  -0.48 log10 CFU/ml carcass rinse Coliforms (compared to immersions chill in chlorinated water) | - pilot-scale paddle-agitated chill tank or cold room - Lab work (carcass halves) | ([Berrang et al., 2008](#_ENREF_13))(USA) |
| Air chilling | -0.26 log 10 CFU/ ml carcass rinse *E. coli* (compared to immersions chill in chlorinated water)  -0.25 log 10 CFU/ ml carcass rinse Coliforms (compared to immersions chill in chlorinated water) | - 2-step Air chilling (-7.7 to -5.5°C and -4.4 to -1.1°C) - Chlorinated water? (not approved in the EU) | ([Sanchez et al., 2002](#_ENREF_63))(USA) |
| Air chilling | 0.04 log10 CFU/ml carcass rinse *E. coli* (compared to water immersions chilling)  0.08 log10 CFU/ml carcass rinse Coliforms (compared to water immersions chilling) | - Spray disinfection with cetylpyridinium chloride before chilling (not approved in the EU) | ([Zhang et al., 2011](#_ENREF_73))(USA) |
| Steam treatment | Treatment time 10s  1.72 log10 CFU/cm2 *E. coli* K12  Treatment time 12s  2.26 log10 CFU/cm2 *E. coli* K12  Treatment time 20s  2.83 log10 CFU/cm2 *E. coli* K12 | - pilot steam cabinet - shrinking skin and change in color | ([James et al., 2007](#_ENREF_34))(UK) |
| Steam or hot water in combination with rapid cooling, chilling or freezing | Steam treatment  1.66 to 3.19 log10 CFU/cm2 *E. coli* K12  Hot water treatment  1.63 to 2.95 log10 CFU/cm2 *E. coli* K12 | - pilot steam cabinet - shrinking skin and change in color | ([James et al., 2007](#_ENREF_34))(UK) |
| Freeze-chilling | ~0 to 1 log10 CFU/g fillet Enterobacteriaceae (compared to air-chilling) | - Freeze-chilling for 7 days - For transportation of fresh meat to final destination | ([Patsias et al., 2008](#_ENREF_55))(GRE) |
| Crust freezing | 0.2 to 0.3 log10 CFU/ml rinse *E. coli* K12 | - Experimental inoculation trial | ([Chaves et al., 2011](#_ENREF_23))(USA) |
| Chilling with water sprays | 0.48 log10 CFU/g breast skin Coliforms | - Experimental rig | ([Allen et al., 2000a](#_ENREF_2))(UK) |
| Chilling with chlorinated water sprays | 50ppm chlorine  0.26 log10 CFU/g breast skin Coliforms  250ppm chlorine  0.62 log10 CFU/g breast skin Coliforms | - Experimental rig - Chlorinated water (not approved in the EU) | ([Allen et al., 2000a](#_ENREF_2))(UK) |
| Chilling in tap water | 1.1 log10 CFU/ml carcass rinse *E. coli*  0.9 log10 CFU/ml carcass rinse Coliforms | - Lab work (30 min, 1°C simulated chill) | ([Dickens et al., 2000](#_ENREF_24))(USA) |
| Renewal of chilling water | Renewal after 8h  1.25 log10 CFU/g carcass *E. coli*  1.22 log10 CFU/g carcass Enterobacteriaceae  1.35 log10 CFU/g carcass Coliforms  Renewal after 16h  1.34 log10 CFU/g carcass *E. coli*  1.05 log10 CFU/g carcass Enterobacteriaceae  1.25 log10 CFU/g carcass Coliforms | - Pooled samples (5 carcasses x 5g/carcass) - free residual chlorine > 2 ppm | ([Souza et al., 2012](#_ENREF_68))(BRA) |
| Immersions chilling with 25ppm chlorinated water | With visible fecal contamination  1.16 log10 CFU/ml carcass rinse *E. coli*  1.02 log10 CFU/ml carcass rinse Enterobacteriaceae  1.23 log10 CFU/ml carcass rinse Coliforms  Without visible fecal contamination  0.89 log10 CFU/ml carcass rinse *E. coli*  0.36 log10 CFU/ml carcass rinse Enterobacteriaceae  0.61 log10 CFU/ml carcass rinse Coliforms | - Chlorinated water (not approved in the EU) | ([Jimenez et al., 2003](#_ENREF_37))(ARG) |
| Chilling with sodium hypochlorite (SH) | -0.14 to 0.71 log10 CFU/100ml carcass rinse *E. coli* (compared to water chiller)  -0.13 to 076 log10 CFU/100ml carcass rinse Coliforms (compared to water chiller) | - Feathered and featherless broiler, single- and triple-scald tank - Different sampling methods in the trials - Chlorinated water (not approved in the EU) | ([Buhr et al., 2005b](#_ENREF_17))(USA) |
| Chilling with sodium hypochlorite (SH) | 0.9 log10 CFU/ml carcass rinse *E. coli*  1.15 log10 CFU/ml carcass rinse Coliforms | - Lab work (Carcass halves: feces contaminated + controls) - Chlorinated water (not approved in the EU) | ([Cason et al., 2004a](#_ENREF_19))(USA) |
| Chilling with sodium hypochlorite (SH) | 10-50ppm  1.59 log10 CFU/25cm2 carcass Coliforms  50-70ppm  1.46 log10 CFU/25cm2 carcass Coliforms | - middle-size processing plant - Chlorinated water (not approved in the EU) | ([Kameyama et al., 2012](#_ENREF_38))(JPN) |
| Chilling with sodium hypochlorite (SH) | 1.4 log10 CFU/ml carcass rinse *E. coli*  1.2 log10 CFU/ml carcass rinse Coliforms | - cotton plugs were inserted into the cloaca during bleeding - not established on-line - Chlorinated water (not approved in the EU) | ([Northcutt et al., 2003a](#_ENREF_48))(USA) |
| Chilling with sodium hypochlorite (SH) | -0.2 log10 CFU/100ml carcass rinse *E. coli* (compared to tap water) | - Chlorinated water (not approved in the EU) | ([Russell and Axtell, 2005](#_ENREF_61))(USA) |
| Chilling with Monochloramine (MON) | 0.9 log10 CFU/100ml carcass rinse *E. coli* (compared to tap water) | - Chlorinated water (not approved in the EU) | ([Russell and Axtell, 2005](#_ENREF_61))(USA) |
| Chlorine (Cl2) immersions chiller | 0.3 log10 CFU/ml carcass rinse *E. coli*  0.4 log10 CFU/ml carcass rinse Coliforms | - Chlorinated water (not approved in the EU) | ([Stopforth et al., 2007](#_ENREF_70))(USA) |
| Acidified chlorine (ClO2-Cl2) immersions chiller | 0.8 log10 CFU/ml carcass rinse *E. coli*  1.0 log10 CFU/ml carcass rinse Coliforms | - Chlorinated water (not approved in the EU) | ([Stopforth et al., 2007](#_ENREF_70)) (USA) |
| Cooled and Chlorinated Chiller Water (2-9°C) | 1.5 log10 CFU/25cm2 carcass Coliforms (compared to 10-19°C chiller water temperature) | - middle-size processing plant - Chlorinated water (not approved in the EU) | ([Kameyama et al., 2012](#_ENREF_38))(JPN) |
| Chilling water with 2% Protecta II (herbal extract on an NaCl carrier) | 2.0 log10 CFU/ml carcass rinse *E. coli*  2.64 log10 CFU/ml carcass rinse Coliforms | - Lab work (30 min, 1°C simulated chill) | ([Dickens et al., 2000](#_ENREF_24))(USA) |
| Post-chill dip in acidified sodium chlorite (ASC) | 1.22 log10 CFU/ml carcass rinse *E. coli* (2days trial)  1.74 log10 CFU/ml carcass rinse *E. coli* (4days trial) | - Chlorinated water (not approved in the EU) | ([Oyarzabal et al., 2004](#_ENREF_52))(USA) |
| Post-chill wash | 0.3 log10 CFU/ml carcass rinse *E. coli*  0.2 log10 CFU/ml carcass rinse Coliforms | - Chlorinated water (not approved in the EU) | ([Stopforth et al., 2007](#_ENREF_70))(USA) |
| Spray wash with electrolyzed water (EO) | 5 sec spray  0.37 log10 MPN/cm2 Coliforms (60 mg/L compared to water)  0.31 log10 MPN/cm2 Coliforms (30 mg/L compared to water)  15 sec spray  0.64 log10 MPN/cm2 Coliforms (60 mg/L compared to water)  0.56 log10 MPN/cm2 Coliforms (30 mg/L compared to water) | - snow-hill yellow-feather breed - 105 days of age | ([Wang et al., 2018](#_ENREF_71))(CHN) |
| Salt application | Pre-chill application:  1.39 log10 CFU/ml carcass rinse *E. coli* (compared to chilled control)  0.66 log10 CFU/ml carcass rinse Coliforms (compared to chilled control)  Post-chill application:  1.77 log10 CFU/ml carcass rinse *E. coli* (compared to chilled control)  1.25 log10 CFU/ml carcass rinse Coliforms (compared to chilled control) | - Kosher salt: larger grain size compared with regular table salt (no dissolving when coating meat) - Lab work | ([Shin et al., 2012](#_ENREF_65))(USA) |
|  |  |  |  |
| **Packaging** |  |  |  |
| CO2 3-h soluble gas stabilization (SGS) | 0.0 log10 CFU/g drumstick *E. coli*  ~0.2 log10 CFU/g drumstick Coliforms (not signif.) | - MAP: 70% CO2, 15% O2 and 15% N2 | ([Al-Nehlawi et al., 2013](#_ENREF_1))(ESP) |
| MAP | 100% CO2  1.24 log10 CFU *E. coli* (compared to air stored after 14d @ 2°C)  5% O2 + 10% CO2 + 85% N  0.56 log10 CFU *E. coli* (compared to air stored after 14d @ 2°C)  100% O2  0.8 log10 CFU *E. coli* (compared to air stored after 14d @ 2°C) | - *Campylobacter* sp. trails | ([Byrd et al., 2011](#_ENREF_18))(USA) |
| MAP | 10:90 N2:CO2  0.14 log10 CFU/cm2 fillet Enterobacteriaceae (day1, compared to air control)  2.62 log10 CFU/cm2 fillet Enterobacteriaceae (day5, compared to air control)  1.81 log10 CFU/cm2 fillet Enterobacteriaceae (day7, compared to air control)  30:70 N2:CO2  -0.25 log10 CFU/cm2 fillet Enterobacteriaceae (day1, compared to air control)  1.78 log10 CFU/cm2 fillet Enterobacteriaceae (day5, compared to air control)  2.07 log10 CFU/cm2 fillet Enterobacteriaceae (day7, compared to air control)  50:50 N2:CO2  0.33 log10 CFU/cm2 fillet Enterobacteriaceae (day1, compared to air control)  1.68 log10 CFU/cm2 fillet Enterobacteriaceae (day5, compared to air control)  2.02 log10 CFU/cm2 fillet Enterobacteriaceae (day7, compared to air control)  70:30 N2:CO2  -0.22 log10 CFU/cm2 fillet Enterobacteriaceae (day1, compared to air control)  1.10 log10 CFU/cm2 fillet Enterobacteriaceae (day5, compared to air control)  1.97 log10 CFU/cm2 fillet Enterobacteriaceae (day7, compared to air control)  90:10 N2:CO2  0.16 log10 CFU/cm2 fillet Enterobacteriaceae (day1, compared to air control)  -0.27 log10 CFU/cm2 fillet Enterobacteriaceae (day5, compared to air control)  0.80 log10 CFU/cm2 fillet Enterobacteriaceae (day7, compared to air control)  80:20 O2:N2  0.36 log10 CFU/cm2 fillet Enterobacteriaceae (day1, compared to air control)  0.03 log10 CFU/cm2 fillet Enterobacteriaceae (day5, compared to air control)  0.70 log10 CFU/cm2 fillet Enterobacteriaceae (day7, compared to air control)  40:30:30 CO2:O2:N2  0.30 log10 CFU/cm2 fillet Enterobacteriaceae (day1, compared to air control)  1.66 log10 CFU/cm2 fillet Enterobacteriaceae (day5, compared to air control)  2.37 log10 CFU/cm2 fillet Enterobacteriaceae (day7, compared to air control) | - Inoculation trails with *Campylobacter* sp. | ([Meredith et al., 2014](#_ENREF_44))(IRE, MLT, NOR, UK) |
| MAP | ~2 to 2.5 log10 CFU/g fillet Enterobacteriaceae (compared to aerobically packed, at day 9) | - 70% N2–30%CO2 | ([Patsias et al., 2008](#_ENREF_55))(GRE) |
| Water Extract of Sumac (8% WES) | ~1.0 log10 CFU/g Coliforms (compared to distilled water)  3d storage time @ 3°C  ~0.5 log10 CFU/g Coliforms (compared to distilled water) | - broiler wing model - sampling after decontamination for 10 min | ([Gulmez et al., 2006](#_ENREF_27))(TUR) |
| Lactic Acid (2% LA) | ~1.7 log10 CFU/g Coliforms (compared to distilled water)  3d storage time @ 3°C  ~2 log10 CFU/g Coliforms (compared to distilled water) | - broiler wing model - sampling after decontamination for 10 min - Lactic acid not approved in the EU | ([Gulmez et al., 2006](#_ENREF_27))(TUR) |
| Active packaging with carvacrol (CAR) | 3% CAR, storage 72h@23°C  6.8 log10 CFU/g chicken breast *E. coli* O157:H7  1.5% CAR, storage 72h@23°C  4.3 log10 CFU/g chicken breast *E. coli* O157:H7  0.5% CAR, storage 72h@23°C  2.8 log10 CFU/g chicken breast *E. coli* O157:H7  3% CAR, storage 72h@4°C  3.0 log10 CFU/g chicken breast *E. coli* O157:H7  1.5% CAR, storage 72h@4°C  3.0 log10 CFU/g chicken breast *E. coli* O157:H7  0.5% CAR, storage 72h@4°C  1.0 log10 CFU/g chicken breast *E. coli* O157:H7 | - Experimental contamination trial - Edible apple films - Not approved in the EU | ([Ravishankar et al., 2009](#_ENREF_56)) (USA) |
| Active packaging with cinnamaldehyde (CIN) | 3% CIN, storage 72h@23°C  5.2 log10 CFU/g chicken breast *E. coli* O157:H7  1.5% CIN, storage 72h@23°C  3.8 log10 CFU/g chicken breast *E. coli* O157:H7  0.5% CIN, storage 72h@23°C  1.8 log10 CFU/g chicken breast *E. coli* O157:H7  3% CIN, storage 72h@4°C  1.2 log10 CFU/g chicken breast *E. coli* O157:H7  1.5% CIN, storage 72h@4°C  1.2 log10 CFU/g chicken breast *E. coli* O157:H7  0.5% CIN, storage 72h@4°C  0.2 log10 CFU/g chicken breast *E. coli* O157:H7 | - Experimental contamination trial - Edible apple films - Not approved in the EU | ([Ravishankar et al., 2009](#_ENREF_56)) (USA) |
| Active packaging with ovotransferrin | Ovotransferrin  0.88 log10 CFU/g chicken breast *E. coli* (compared to control after 7d @5°C)  Ovotransferrin+EDTA (5mM)  2.66 log10 CFU/g chicken breast *E. coli* (compared to control after 7d @5°C) | - Experimental contamination trial - Kappa-carrageenan-based films - Not approved in the EU - Reduction due to EDTA only was 1.4 log10 CFU/g chicken breast *E. coli* | ([Seol et al., 2009](#_ENREF_64)) (KOR) |
| Active packaging with potassium sorbat | Potassium sorbat  0.64 log10 CFU/g chicken breast *E. coli* (compared to control after 7d @5°C)  Potassium sorbat+EDTA (5mM)  2.07 log10 CFU/g chicken breast *E. coli* (compared to control after 7d @5°C) | - Experimental contamination trial - Kappa-carrageenan-based films - Not approved in the EU - Reduction due to EDTA only was 1.4 log10 CFU/g chicken breast *E. coli* | ([Seol et al., 2009](#_ENREF_64)) (KOR) |
| High-intensitiy pulsed light | 2 sec treatment time, uncovered  1.26 log10 CFU/g chicken skin *E. coli*  30 sec treatment time, uncovered  1.51 log10 CFU/g chicken skin *E. coli* | - Experimental contamination trial - Use of various plastic films resulted in less reduction of *E. coli* | ([Haughton et al., 2011](#_ENREF_28))(IRE) |
|  |  |  |  |
| **Equipment/Others** |  |  |  |
| Peracetic Acid and Quarternary Ammonium Disinfectants as a Part of Sanitary Treatment | ~5.2 log10 CFU Coliforms | - Swabbed places: Shackling hooks, Eviscerator, Cooling tank, Shoots, Convey belts, Saws, (Plastic) Boards, Knives, Injector | ([Kašková et al., 2007](#_ENREF_39))(SVK) |
| sodium hypochlorite (SH) for sanitizing stainless steel | 100 mg/l, 10 min: 116.5 cells /field area *E. coli*  200 mg/l, 10 min: 117.5 cells /field area *E. coli* | - Lab work - 8.66x10-3 mm2 | ([Rossoni and Gaylarde, 2000](#_ENREF_57))(BRA) |
| peracetic acid for sanitizing stainless steel | 250 mg/l, 10 min: 108.5 cells /field area *E. coli*  1000 mg/l, 10 min: 112.5 cells /field area *E. coli* | - Lab work - 8.66x10-3 mm2 | ([Rossoni and Gaylarde, 2000](#_ENREF_57))(BRA) |
| Transport crate treatment | 15 s pre-wash, 30 s soak, 120 s brush, 15 s main wash (63C), 500 ml 2% Virkon S  5.4 log10 CFU Enterobacteriaceae  15 s pre-wash, 30 s soak (52C), 300 s brush, 20 s main wash (63C)  3.8 log10 CFU Enterobacteriaceae  15 s pre-wash, 30 s soak, 15 s main wash (63C), 500ml 2% Virkon S  3.6 log10 CFU Enterobacteriaceae  15 s pre-wash, 30 s soak in clean water with 01% detergent (55 to 60C), 15 s clean wash (63C)  3.2 log10 CFU Enterobacteriaceae  15 s pre-wash, 30 s soak in clean water with 01% detergent (55 to 60C), 15 s clean wash (63C), wash repeated  4.1 log10 CFU Enterobacteriaceae  Ultrasound  0.8 to 1.4 log10 CFU Enterobacteriaceae | - test rig - ultrasonic treatment effective in conjunction with higher immersion temperatures | ([Allen et al., 2008a](#_ENREF_3); [Allen et al., 2008b](#_ENREF_5))(UK) |
| Conveyor belt hot water treatment | 0.12 to 0.25 log10 CFU/cm2 Enterobacteriaceae | - No significant differences | ([Soares et al., 2014](#_ENREF_66))(BRA) |
| pulsed-plasma gas-discharge (PPGD) system for decontamination of chilled poultry wash water | reduced Escherichia coli NCTC 9001, Campylobacter jejuni ATCC 33560, Campylobacter coli ATCC 33559, Listeria monocytogenes NCTC 9863, Salmonella enterica serovar Enteritidis ATCC 4931, and S. enterica serovar Typhimurium ATCC 14028 populations to nondetectable levels (> 8 log CFU/ml reduction) | - Lab work | ([Rowan et al., 2007](#_ENREF_58))(UK) |
| 254 nm UV irradiation | Stainless steel chips  250 mW/cm2  1, 2, 3 min: 4.38, 4.68, 5.2 log10 CFU/ml *E. coli*  500 mW/cm2  1, 2 min: 4.71, 5.34 log10 CFU/ml *E. coli*  Chicken meat with skin  500 mW/cm2  1, 2, 3 min: 0.17, 0.76, 1.28 log10 CFU/cm2 *E. coli*  Chicken meat without skin  500 mW/cm2  1, 2, 3 min: 0.24, 0.26, 0.93 log10 CFU/cm2 *E. coli* | - Lab work - Stainless steel chips: 2 by 2 cm2 - Chicken breast meat: 5 by 5 by 1 cm3 | ([Kim et al., 2002](#_ENREF_42))(USA) |
| 405-nm light LED array  (180 J/cm2) | Chicken skin: 10 min (300 mW/cm2/s)  ~0.4 log10 CFU *E. coli*  Chicken skin: 20 min (150 mW/cm2/s)  ~0.2 log10 CFU *E. coli*  stainless steel coupons: 10 min (300 mW/cm2/s)  ~0.9 log10 CFU *E. coli*  stainless steel coupons: 20 min (150 mW/cm2/s)  ~0.5 log10 CFU *E. coli* | - Lab work - 2 by 2 cm2 | ([Sommers et al., 2017](#_ENREF_67))(USA) |
|  |  |  |  |

References

Al-Nehlawi, A., Saldo, J., Vega, L.F., Guri, S., 2013. Effect of high carbon dioxide atmosphere packaging and soluble gas stabilization pre-treatment on the shelf-life and quality of chicken drumsticks. Meat science 94, 1-8.

Allen, V.M., Burton, C.H., Corry, J.E., Mead, G.C., Tinker, D.B., 2000a. Investigation of hygiene aspects during air chilling of poultry carcases using a model rig. British poultry science 41, 575-583.

Allen, V.M., Burton, C.H., Wilkinson, D.J., Whyte, R.T., Harris, J.A., Howell, M., Tinker, D.B., 2008a. Evaluation of the performance of different cleaning treatments in reducing microbial contamination of poultry transport crates. British poultry science 49, 233-240.

Allen, V.M., Corry, J.E., Burton, C.H., Whyte, R.T., Mead, G.C., 2000b. Hygiene aspects of modern poultry chilling. Int J Food Microbiol 58, 39-48.

Allen, V.M., Whyte, R.T., Burton, C.H., Harris, J.A., Lovell, R.D., Atterbury, R.J., Tinker, D.B., 2008b. Effect of ultrasonic treatment during cleaning on the microbiological condition of poultry transport crates. British poultry science 49, 423-428.

Barbut, S., Moza, L.F., Nattress, F., Dilts, B., Gill, C.O., 2009. The microbiological conditions of air- or water-chilled carcasses produced at the same poultry packing plant. J Appl Poultry Res 18, 501-507.

Barreiro, F.R., Baraldi-Artoni, S.M., Pinto, F.R., Barbosa, M.M., Barbosa, J.C., Amaral, L.A., 2012. Influence of chlorine added to drinking water during the preslaughter feed withdrawal on microbiology and morphology of the broiler gastrointestinal tract. Poult Sci 91, 2778-2784.

Bartenfeld, L.N., Fletcher, D.L., Northcutt, J.K., Bourassa, D.V., Cox, N.A., Buhr, R.J., 2014. The effect of high-level chlorine carcass drench on the recovery of Salmonella and enumeration of bacteria from broiler carcasses. Poult Sci 93, 2893-2899.

Berrang, M.E., Bailey, J.S., 2009. On-line brush and spray washers to lower numbers of Campylobacter and Escherichia coli and presence of Salmonella on broiler carcasses during processing. J Appl Poultry Res 18, 74-78.

Berrang, M.E., Buhr, R.J., Cason, J.A., Dickens, J.A., 2002. Microbiological consequences of skin removal prior to evisceration of broiler carcasses. Poult Sci 81, 134-138.

Berrang, M.E., Dickens, J.A., Musgrove, M.T., 2000. Effects of hot water application after defeathering on the levels of Campylobacter, coliform bacteria, and Escherichia coli on broiler carcasses. Poult Sci 79, 1689-1693.

Berrang, M.E., Meinersmann, R.J., Buhr, R.J., Reimer, N.A., Philips, R.W., Harrison, M.A., 2003. Presence of Campylobacter inthe respiratory tract of broiler carcasses before and after commercial scalding. Poult Sci 82, 1995-1999.

Berrang, M.E., Meinersmann, R.J., Smith, D.P., Zhuang, H., 2008. The effect of chilling in cold air or ice water on the microbiological quality of broiler carcasses and the population of Campylobacter. Poult Sci 87, 992-998.

Berrang, M.E., Windham, W.R., Meinersmann, R.J., 2011. Campylobacter, Salmonella, and Escherichia coli on broiler carcasses subjected to a high pH scald and low pH postpick chlorine dip. Poult Sci 90, 896-900.

Buhr, R.J., Berrang, M.E., Cason, J.A., 2003. Bacterial recovery from breast skin of genetically feathered and featherless broiler carcasses immediately following scalding and picking. Poult Sci 82, 1641-1647.

Buhr, R.J., Berrang, M.E., Cason, J.A., Bourassa, D.V., 2005a. Recovery of bacteria from broiler carcass respiratory tracts before and after immersion scalding. Poult Sci 84, 1769-1773.

Buhr, R.J., Bourassa, D.V., Northcutt, J.K., Hinton, A., Jr., Ingram, K.D., Cason, J.A., 2005b. Bacteria recovery from genetically feathered and featherless broiler carcasses after immersion chilling. Poult Sci 84, 1499-1504.

Byrd, J.A., Sams, A.R., Hargis, B.M., Caldwell, D.J., 2011. Effect of selected modified atmosphere packaging on Campylobacter survival in raw poultry. Poult Sci 90, 1324-1328.

Cason, J.A., Berrang, M.E., Buhr, R.J., Cox, N.A., 2004a. Effect of prechill fecal contamination on numbers of bacteria recovered from broiler chicken carcasses before and after immersion chilling. J Food Prot 67, 1829-1833.

Cason, J.A., Buhr, R.J., Hinton, A., Jr., 2001. Unheated water in the first tank of a three-tank broiler scalder. Poult Sci 80, 1643-1646.

Cason, J.A., Hinton, A., Jr., Buhr, R.J., 2004b. Impact of feathers and feather follicles on broiler carcass bacteria. Poult Sci 83, 1452-1455.

Cason, J.A., Hinton, A., Jr., Ingram, K.D., 2000. Coliform, Escherichia coli, and salmonellae concentrations in a multiple-tank, counterflow poultry scalder. J Food Prot 63, 1184-1188.

Chaves, B.D., Han, I.Y., Dawson, P.L., Northcutt, J.K., 2011. Survival of artificially inoculated Escherichia coli and Salmonella Typhimurium on the surface of raw poultry products subjected to crust freezing. Poult Sci 90, 2874-2878.

Dickens, J.A., Berrang, M.E., Cox, N.A., 2000. Efficacy of an herbal extract on the microbiological quality of broiler carcasses during a simulated chill. Poult Sci 79, 1200-1203.

Giombelli, A., Gloria, M.B., 2014. Prevalence of Salmonella and Campylobacter on broiler chickens from farm to slaughter and efficiency of methods to remove visible fecal contamination. J Food Prot 77, 1851-1859.

Giombelli, A., Hammerschmitt, D., Cerutti, M.F., Chiarini, E., Landgraf, M., Franco, B.D., Destro, M.T., 2015. High pressure spray with water shows similar efficiency to trimming in controlling microorganisms on poultry carcasses. Poult Sci 94, 2589-2595.

Gulmez, M., Oral, N., Vatansever, L., 2006. The effect of water extract of sumac (Rhus coriaria L.) and lactic acid on decontamination and shelf life of raw broiler wings. Poult Sci 85, 1466-1471.

Haughton, P.N., Lyng, J.G., Morgan, D.J., Cronin, D.A., Fanning, S., Whyte, P., 2011. Efficacy of high-intensity pulsed light for the microbiological decontamination of chicken, associated packaging, and contact surfaces. Foodborne pathogens and disease 8, 109-117.

Hinton, A., Ingram, K.D., 2000. Use of oleic acid to reduce the population of the bacterial flora of poultry skin. Journal of food protection 63, 1282-1286.

Hinton, A., Jr., Buhr, R.J., Ingram, K.D., 2000. Reduction of Salmonella in the crop of broiler chickens subjected to feed withdrawal. Poult Sci 79, 1566-1570.

Hinton, A., Jr., Ingram, K.D., 2005. Microbicidal activity of tripotassium phosphate and fatty acids toward spoilage and pathogenic bacteria associated with poultry. J Food Prot 68, 1462-1466.

Hinton, A., Jr., Ingram, K.D., 2006. Antimicrobial activity of potassium hydroxide and lauric acid against microorganisms associated with poultry processing. J Food Prot 69, 1611-1615.

Hinton, A., Northcutt, J.K., Cason, J.A., Smith, D.P., Ingram, K.D., 2007. Bacterial Populations of broiler carcasses washed in mixtures of potassium hydroxide and lauric acid. J Appl Poultry Res 16, 387-391.

James, C., James, S.J., Hannay, N., Purnell, G., Barbedo-Pinto, C., Yaman, H., Araujo, M., Gonzalez, M.L., Calvo, J., Howell, M., Corry, J.E., 2007. Decontamination of poultry carcasses using steam or hot water in combination with rapid cooling, chilling or freezing of carcass surfaces. Int J Food Microbiol 114, 195-203.

James, W.O., Prucha, J.C., Brewer, R.L., Williams, W.O., Jr., Christensen, W.A., Thaler, A.M., Hogue, A.T., 1992. Effects of countercurrent scalding and postscald spray on the bacteriologic profile of raw chicken carcasses. Journal of the American Veterinary Medical Association 201, 705-708.

Jimenez, S.M., Destefanis, P., Salsi, M.S., Tiburzi, M.C., Pirovani, M.E., 2005. Predictive model for reduction of Escherichia coli during acetic acid decontamination of chicken skin. J Appl Microbiol 99, 829-835.

Jimenez, S.M., Tiburzi, M.C., Salsi, M.S., Pirovani, M.E., Moguilevsky, M.A., 2003. The role of visible faecal material as a vehicle for generic Escherichia coli, coliform, and other enterobacteria contaminating poultry carcasses during slaughtering. Journal of applied microbiology 95, 451-456.

Kameyama, M., Chuma, T., Nishimoto, T., Oniki, H., Yanagitani, Y., Kanetou, R., Gotou, K., Shahada, F., Iwata, H., Okamoto, K., 2012. Effect of Cooled and Chlorinated Chiller Water on Campylobacter and Coliform Counts on Broiler Carcasses during Chilling at a Middle-Size Poultry Processing Plant. Journal of Veterinary Medical Science 74, 129-133.

Kašková, A., Ondrasovicova, O., Vargova, M., Ondrasovic, M., Venglovsky, J., 2007. Application of peracetic acid and quarternary ammonium disinfectants as a part of sanitary treatment in a poultry house and poultry processing plant. Zoonoses and public health 54, 125-130.

Kemp, G.K., Aldrich, M.L., Guerra, M.L., Schneider, K.R., 2001. Continuous online processing of fecal- and ingesta- contaminated poultry carcasses using an acidified sodium chlorite antimicrobial intervention. Journal of food protection 64, 807-812.

Kemp, G.K., Aldrich, M.L., Waldroup, A.L., 2000. Acidified sodium chlorite antimicrobial treatment of broiler carcasses. J Food Prot 63, 1087-1092.

Kim, T., Silva, J.L., Chen, T.C., 2002. Effects of UV irradiation on selected pathogens in peptone water and on stainless steel and chicken meat. J Food Prot 65, 1142-1145.

Lansini, V., Maia, D.S.V., da Fontoura Prates, D., de Lima, A.S., da Silva, W.P., 2017. Antibacterial activity of Timsen((R)) (n-alkyl dimethyl benzyl ammonium chloride-40%) in scalding and precooling water in poultry slaughterhouses. Journal of food science and technology 54, 2607-2612.

Meredith, H., Valdramidis, V., Rotabakk, B.T., Sivertsvik, M., McDowell, D., Bolton, D.J., 2014. Effect of different modified atmospheric packaging (MAP) gaseous combinations on Campylobacter and the shelf-life of chilled poultry fillets. Food microbiology 44, 196-203.

Mulder, R.W., Bolder, N.M., 1981. The effect of different bird washers on the microbiological quality of broiler carcasses. The Veterinary quarterly 3, 124-130.

Musgrove, M.T., Cason, J.A., Fletcher, D.L., Stern, N.J., Cox, N.A., Bailey, J.S., 1997. Effect of cloacal plugging on microbial recovery from partially processed broilers. Poult Sci 76, 530-533.

Northcutt, J., Smith, D., Ingram, K.D., Hinton, A., Jr., Musgrove, M., 2007. Recovery of bacteria from broiler carcasses after spray washing with acidified electrolyzed water or sodium hypochlorite solutions. Poult Sci 86, 2239-2244.

Northcutt, J.K., Berrang, M.E., Dickens, J.A., Fletcher, D.L., Cox, N.A., 2003a. Effect of broiler age, feed withdrawal, and transportation on levels of coliforms, Campylobacter, Escherichia coli and Salmonella on carcasses before and after immersion chilling. Poult Sci 82, 169-173.

Northcutt, J.K., Buhr, R.J., Berrang, M.E., Fletcher, D.L., 2003b. Effects of replacement finisher feed and length of feed withdrawal on broiler carcass yield and bacteria recovery. Poult Sci 82, 1820-1824.

Northcutt, J.K., McNeal, W.D., Ingram, K.D., Buhr, R.J., Fletcher, D.L., 2008. Microbial recovery from genetically featherless broiler carcasses after forced cloacal fecal expulsion. Poult Sci 87, 2377-2381.

Notermans, S., Kampelmacher, E.H., 1975. Heat destruction of some bacterial strains attached to broiler skin. British poultry science 16, 351-361.

Oyarzabal, O.A., Hawk, C., Bilgili, S.F., Warf, C.C., Kemp, G.K., 2004. Effects of postchill application of acidified sodium chlorite to control Campylobacter spp. and Escherichia coli on commercial broiler carcasses. J Food Prot 67, 2288-2291.

Pacholewicz, E., Lipman, L.J., Swart, A., Havelaar, A.H., Heemskerk, W.J., 2016. Pre-scald brushing for removal of solids and associated broiler carcass bacterial contamination. Poult Sci 95, 2979-2985.

Patrick, T.E., Goodwin, T.L., Collins, J.A., Wyche, R.C., Love, B.E., 1972. Steam versus hot-water scalding in reducing bacterial loads on the skin of commercially processed poultry. Applied microbiology 23, 796-798.

Patsias, A., Badeka, A.V., Savvaidis, I.N., Kontominas, M.G., 2008. Combined effect of freeze chilling and MAP on quality parameters of raw chicken fillets. Food microbiology 25, 575-581.

Ravishankar, S., Zhu, L., Olsen, C.W., McHugh, T.H., Friedman, M., 2009. Edible apple film wraps containing plant antimicrobials inactivate foodborne pathogens on meat and poultry products. Journal of food science 74, M440-445.

Rossoni, E.M., Gaylarde, C.C., 2000. Comparison of sodium hypochlorite and peracetic acid as sanitising agents for stainless steel food processing surfaces using epifluorescence microscopy. Int J Food Microbiol 61, 81-85.

Rowan, N.J., Espie, S., Harrower, J., Anderson, J.G., Marsili, L., MacGregor, S.J., 2007. Pulsed-plasma gas-discharge inactivation of microbial pathogens in chilled poultry wash water. J Food Prot 70, 2805-2810.

Rubio-Garcia, M.E., Rubio-Lozano, M.S., Ponce-Alquicira, E., Rosario-Cortes, C., Nava, G.M., Castaneda-Serrano, M.P., 2015. Improving appearance and microbiologic quality of broiler carcasses with an allostatic modulator. Poult Sci 94, 1957-1963.

Russell, S.M., 2008. The effect of an acidic, copper sulfate-based commercial sanitizer on indicator, pathogenic, and spoilage bacteria associated with broiler chicken carcasses when applied at various intervention points during poultry processing. Poult Sci 87, 1435-1440.

Russell, S.M., Axtell, S.P., 2005. Monochloramine versus sodium hypochlorite as antimicrobial agents for reducing populations of bacteria on broiler chicken carcasses. J Food Prot 68, 758-763.

Russell, S.M., Walker, J.M., 1997. The effect of evisceration on visible contamination and the microbiological profile of fresh broiler chicken carcasses using the Nu-Tech Evisceration System or the conventional Streamlined Inspection System. Poult Sci 76, 780-784.

Sanchez, M.X., Fluckey, W.M., Brashears, M.M., McKee, S.R., 2002. Microbial profile and antibiotic susceptibility of Campylobacter spp. and Salmonella spp. in broilers processed in air-chilled and immersion-chilled environments. J Food Prot 65, 948-956.

Seol, K.H., Lim, D.G., Jang, A., Jo, C., Lee, M., 2009. Antimicrobial effect of kappa-carrageenan-based edible film containing ovotransferrin in fresh chicken breast stored at 5 degrees C. Meat science 83, 479-483.

Shin, D., Kakani, G., Molina, V.A., Regenstein, J.M., Choe, H.S., Sanchez-Plata, M.X., 2012. Effect of kosher salt application on microbial profiles of poultry carcasses. Poult Sci 91, 3247-3252.

Soares, V.M., Pereira, J.G., Zanette, C.M., Nero, L.A., Pinto, J.P., Barcellos, V.C., Bersot, L.S., 2014. Cleaning conveyor belts in the chicken-cutting area of a poultry processing plant with 45 degrees c water. J Food Prot 77, 496-498.

Sommers, C., Gunther, N.W.t., Sheen, S., 2017. Inactivation of Salmonella spp., pathogenic Escherichia coli, Staphylococcus spp., or Listeria monocytogenes in chicken purge or skin using a 405-nm LED array. Food microbiology 64, 135-138.

Souza, L.C., Jr., Pereira, J.G., Spina, T.L., Izidoro, T.B., Oliveira, A.C., Pinto, J.P., 2012. Microbiological evaluation of chicken carcasses in an immersion chilling system with water renewal at 8 and 16 hours. J Food Prot 75, 973-975.

Stanley, V.G., Hutchinson, D.M., Reine, A.H., Corrier, D., Hinton, A.A., Jr., 1992. Magnesium sulfate effects on coliform bacteria reduction in the intestines, ceca, and carcasses of broiler chickens. Poult Sci 71, 76-80.

Stopforth, J.D., O'Connor, R., Lopes, M., Kottapalli, B., Hill, W.E., Samadpour, M., 2007. Validation of individual and multiple-sequential interventions for reduction of microbial populations during processing of poultry carcasses and parts. J Food Prot 70, 1393-1401.

Wang, H., Qi, J., Duan, D., Dong, Y., Xu, X., Zhou, G., 2018. Combination of a novel designed spray cabinet and electrolyzed water to reduce microorganisms on chicken carcasses. Food Control 86, 200-206.

Whyte, P., Collins, J.D., McGill, K., Monahan, C., O'Mahony, H., 2001. Quantitative investigation of the effects of chemical decontamination procedures on the microbiological status of broiler carcasses during processing. J Food Prot 64, 179-183.

Zhang, L., Jeong, J.Y., Janardhanan, K.K., Ryser, E.T., Kang, I., 2011. Microbiological quality of water immersion-chilled and air-chilled broilers. J Food Prot 74, 1531-1535.
